# Non-invasive hemoglobin estimation with outcome prediction via deep learning analysis of ECG-derived cardiac micro-dynamics

**DOI:** 10.1007/s11739-025-04209-6

**Published:** 2025-12-02

**Authors:** Chung-Chi Yang, Chin-Sheng Lin, Chin Lin, Wei-Ting Liu, Zih-Yin Lai, Yung-Jen Chuang

**Affiliations:** 1 Division of Cardiovascular Medicine, Taoyuan Armed Forces General Hospital, Taoyuan, 32551 Taiwan, ROC; 2 Division of Cardiology, Department of Internal Medicine, Tri-Service General Hospital, National Defense Medical University, Taipei, Taiwan, ROC; 3 School of Medicine, National Tsing Hua University, Hsinchu, 300044 Taiwan, ROC; 4 Institute of Bioinformatics and Structural Biology, National Tsing Hua University, Hsinchu, 300044 Taiwan, ROC; 5 Medical Technology Education Center, College of Medicine, National Defense Medical University, Taipei, Taiwan, ROC; 6 Departments of Artificial Intelligence of Things, Tri-Service General Hospital, National Defense Medical University, Taipei, Taiwan, ROC; 7 College of Public Health, National Defense Medical University, Taipei, Taiwan, ROC

**Keywords:** Anemia detection, Deep learning model, Electrocardiogram, Heart rate variability, Prognostic value

## Abstract

**Background:**

Anemia is a global health issue, especially in resource-limited areas, where traditional hemoglobin (Hb) testing is invasive and costly. This study aimed to develop an electrocardiogram–hemoglobin (ECG-Hb) deep learning model (DLM) for detecting anemia and assess its impact on all-cause mortality and new-onset heart failure.

**Methods:**

This retrospective study analyzed ECGs and corresponding Hb levels from two hospitals. The DLM was trained on 388,166 ECGs from 187,202 patients and tested on 24,279 and 29,247 patients in internal and external sets, respectively. Anemia was defined as moderate (Hb ≤ 10 g/dL) or severe (Hb ≤ 8 g/dL). Diagnostic performance was evaluated using receiver operating characteristic (ROC) curve analysis, and an 8-year follow-up assessed mortality and heart failure risk with Cox regression.

**Results:**

The areas under the ROC curves (AUCs) for detecting moderate-to-severe anemia were 0.8545 (internal) and 0.8243 (external), with sensitivities of 65.9% and 71.0%, and specificities of 84.8% and 77.4%, respectively. ECG-Hb performed better in detecting severe anemia (AUC = 0.9038/0.8766) than in mild anemia. Pearson correlations between ECG-Hb and Hb were 0.56 (internal) and 0.53 (external). Key ECG features, including heart rate variability, significantly influenced ECG-Hb. Patients with severely low ECG-Hb had higher risks of mortality (hazard ratio [HR]: 1.71, 95% confidence interval [CI]: 1.42–2.06) and heart failure (HR: 2.47, 95% CI: 2.07–2.94) compared to those with standard ECG-Hb levels.

**Conclusion:**

The ECG-Hb DLM offers strong diagnostic and prognostic potential for anemia and cardiovascular risks, making it a valuable, non-invasive screening tool in low-resource settings.

**Supplementary Information:**

The online version contains supplementary material available at 10.1007/s11739-025-04209-6.

## Introduction

Anemia stands as a pressing global health concern, affecting over 800 million individuals, predominantly in resource-limited regions [1]. The World Health Organization (WHO) has proactively outlined a comprehensive strategy to expedite the mitigation of anemia [2]. Anemia can be triggered by a myriad of acute and chronic conditions, encompassing nutritional deficiencies due to subpar diets or impaired nutrient absorption, infections, gastrointestinal bleeding, malignancies, and chronic renal diseases, among others [3]. Patients may not exhibit symptoms in instances of moderate or insidious anemia. Consequently, diagnosis with only patient histories and examinations becomes easier once the condition is uncompensated and complications occur [4]. Anemia can escalate the risk of cardiovascular diseases and mortality [5, 6], necessitating prompt intervention to enhance patient prognosis. While anemia can be diagnosed through laboratory blood tests [7], the invasive nature of the procedure and the substantial costs associated with blood testing equipment signifies that many patients with asymptomatic anemia in various regions remain undiagnosed and untreated [8].

The association between heart rate variability (HRV) and anemia has been extensively reported [9, 10]. Furthermore, numerous studies have indicated that anemia can alter electrocardiogram (ECG) morphology, suggesting that a mismatch between oxygen demand and supply in the myocardium influences ECG patterns [11, 12]. Given the cost-effectiveness and non-invasive nature of electrocardiography, it is particularly suitable for anemia screening in resource-limited regions. Moreover, the evolution of wearable ECG devices has broadened their accessibility and usage in cloud computing. Despite the potential association between HRV, ECG alterations, and anemia, diagnosing anemia based solely on ECG remains challenging.

With the advent of deep learning models (DLMs), these advanced algorithms have been validated to detect conditions, such as acute myocardial infarction, [13, 14] pericarditis, [15] digoxin toxicity, [16] pneumothorax, [17] aortic dissection, [18] thyrotoxic periodic paralysis, [19] and dyskalemias [20], using ECGs, often surpassing the diagnostic capabilities of cardiologists. Furthermore, prior studies have illustrated that artificial intelligence (AI)-enhanced ECGs detect previous history of left ventricular dysfunction even in patients with a normal ejection fraction [21–24]. This capability is not limited to identifying such precursors; AI-empowered ECGs have also pinpointed patient groups at elevated mortality risk, even when their serum potassium concentrations are within the normal range [25]. In addition, when combined with AI, ECGs can estimate "heart age" as an indicator of cardiovascular health[26] and accurately predict short-term mortality [27, 28]. Leveraging AI, ECGs hold the potential to forecast future disease risks in ostensibly healthy individuals and facilitate early preventive measures, thereby mitigating disease risks. Such advancements underscore the significance of primary prevention, health promotion, and targeted protective measures.

A recent investigation pioneered the development of a DLM capable of detecting anemia, achieving an area under the receiver operating characteristic (ROC) curve (AUCs) exceeding 0.85, relying solely on 12-lead ECGs [29]. However, the model's relatively low positive predictive value (PPV) of < 20% and its lack of clarity regarding false-positive findings to precursors of cardiovascular diseases has curtailed its practical applicability. Moreover, no comprehensive analysis has elucidated the underlying mechanisms by which this "black-box" AI model accurately identifies anemia. This study aimed to develop a DLM that employs ECG as a novel biomarker, termed electrocardiogram–hemoglobin (ECG-Hb). Beyond conducting analyses of HRV and ECG patterns to enhance the transparency of the inner workings of AI, we also aimed to explore its implications for all-cause mortality and the onset of heart failure. This study sought to establish guidelines for prospective, large-scale implementation in resource-limited regions for screening purposes.

## Materials and methods

### Data source

This retrospective study was approved by the Ethics Committee of the Tri-Service General Hospital (C202105049) to collect medical data. The study aimed to formulate a DLM and subsequently validate its internal and external efficacies. ECGs were obtained from two hospitals within the Tri-Service General Hospital system: an academic medical institution in Neihu District (Hospital A) and a community-based hospital in Zhongzheng District (Hospital B). The collection period spanned from January 1, 2010 to September 30, 2021. Each ECG was annotated based on the nearest Hb, which varied between 5.0 and 18.0 g/dL. The nearest numerical boundary replaced values outside this range. ECGs that lacked an Hb test within a 12-h window were omitted from the study. No other exclusion criteria were applied.

**Fig. 1:**
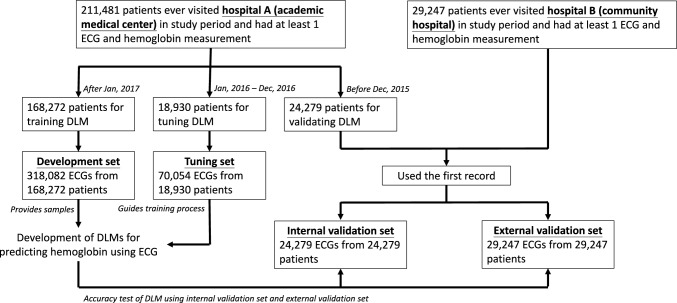
Development, tuning, internal validation, and external validation sets generation and ECG labeling of hemoglobin. Schematic of the data set creation and analysis strategy, which was devised to assure a robust and reliable data set for training, validating, and testing of the network. Once a patient’s data were placed in one of the data sets, the data is used only in that set, avoiding ‘cross-contamination’ among the training, validation, and test data sets. The details of the flow chart and the usage of each data set are described in the Materials and Methods

As shown in Fig. 1, a systematic approach was employed to develop and validate the DLM. Within Hospital A, 211,481 patients presented with at least one paired ECG and Hb for this investigation. Subsequently, 168,272 patients who sought medical care at Hospital A after January 1, 2017 were allocated to the development cohort, contributing 318,082 ECG records for DLM training (mean ECGs per patient: 1.89). The cohort comprising 18,930 patients who visited between January 1, 2016 and December 31, 2016 was designated as the tuning set. Furnishing with 70,054 ECGs was done to facilitate the training process and establish the pivotal diagnostic threshold (mean ECGs per patient: 3.70). Before December 31, 2015, 24,279 patients seeking care were categorized into the internal validation cohort, and their initial ECGs were used for accuracy assessment and subsequent analyses. An external validation set was constructed using data from Hospital B to ascertain the generalizability of the DLM. The validation set encompassed 29,247 patients and adhered to the inclusion criteria identical to those of Hospital A.

### Data collection

Within the ECG dataset, patients were selected based on the presence of at least one standard digital ECG, characterized by a 500 Hz frequency, a duration of 10 s, and a 12-lead configuration obtained in the supine position during the designated study timeframe. Hb levels were determined using cyanide-free sodium lauryl sulfate (SLS) methodology in the central laboratory. The DLM was trained using raw ECG traces. Both quantitative metrics and anomalous findings were derived from the ECG, resulting in 31 diagnostic pattern classifications and 8 continuous ECG measurements. Any missing data within the ECG measurements were addressed by applying multiple imputations [30]. The proportions of missing values were 7.8% for the PR interval, 8.1% for the P-wave axis, and 1.4% for the T-wave axis. Other ECG measurements had no missing values. The 31 clinical diagnostic patterns were discerned from the structured findings and predicted by key terminologies consistent with the Philips system. Based on a previous study, the HRV parameters were calculated based on 10-s raw ECG traces, including standard deviation of normal-to-normal intervals (SDNN), root mean square of successive differences (RMSSD), standard deviation of successive differences (SDSD), and percentage of successive NN intervals that differ by more than 50 ms (pNN50) defined based on a previous study [31]. Anemia classifications were established, with moderate anemia defined by an Hb level of ≤ 10 g/dL and severe anemia characterized by an Hb level of ≤ 8 g/dL.

Disease histories were delineated according to the International Classification of Diseases (ICD), Ninth and Tenth Revision (ICD-9 and ICD-10, respectively). The classifications are as follows: diabetes mellitus (DM, ICD-9 codes 250.x and ICD-10 codes E08.x to E13.x), hypertension (HTN, ICD-9 codes 401.x to 404.x and ICD-10 codes I10.x to I16.x), hyperlipidemia (HLP, ICD-9 codes 272.x and ICD-10 codes E78.x), chronic kidney disease (CKD, ICD-9 codes 585.x and ICD-10 codes N18.x), acute myocardial infarction (AMI, ICD-9 codes 410.x and ICD-10 codes I21.x), stroke (STK, ICD-9 codes 430.x to 438.x and ICD-10 codes I60.x to I63.x), coronary artery disease (CAD, ICD-9 codes 410.x to 414.x, and 429.2, and ICD-10 codes I20.x to I25.x), heart failure (HF, ICD-9 codes 428.x and ICD-10 codes I50.x), atrial fibrillation (AF, ICD-9 codes 427.31 and ICD-10 codes I48.x), and chronic obstructive pulmonary disease (COPD, ICD-9 codes 490.x to 496.x and ICD-10 codes J44.9).

This study focused on complications in all-cause mortality and new-onset heart failure. Survival duration was ascertained using ECG data as a reference point for all-cause mortality. Patient status (deceased or alive) was determined via electronic medical records, which were updated with each hospital interaction. Furthermore, to mitigate potential bias arising from incomplete documentation, data pertaining to live visits were censored during the most recent documented hospital encounter. Relevant ICD codes were used to characterize the incidence of new-onset heart failure. Notably, patients diagnosed with heart failure prior to the ECG date were excluded from the new-onset heart failure complication analysis.

### Deep learning model training

In this study, we employed the previously proposed ECG12Net, an 82-layer convolutional neural network, as referenced in previous studies [32, 33]. For this investigation, we adopted the identical architectural framework to train a DLM to estimate ECG-Hb. The training specifics were consistent with methodologies delineated in prior studies [34–37]. Furthermore, an oversampling procedure was implemented, predicated on the inverse prevalence of each Hb interval, incrementing by 0.5 g/dL, ranging from 5.0 to 18.0 g/dL within the development set.

### Statistical analysis and model performance assessment

Patient demographics were delineated as means with standard deviations, patient counts, or percentages, where appropriate. All statistical evaluations were performed using R software (version 3.4.4) with a predetermined significance threshold of p < 0.05. To juxtapose the actual Hb with the ECG-Hb, scatter plots showing mean differences and standard deviations (Diff), Pearson correlation coefficient (r), and mean absolute error (MAE) were employed. Additionally, the diagnostic efficacy for varying degrees of anemia (moderate and severe) was assessed in both the internal and external validation sets. Metrics, such as area under the receiver operating characteristic curve (AUC), sensitivity (Sens.), specificity (Spec.), positive predictive value (PPV), and negative predictive value (NPV), were elucidated. The optimal operating point was determined based on the maximum Youden index value within the tuning set. Given the disparate Hb distributions in the validation sets, balanced datasets were curated for each set, ensuring uniform case numbers across Hb values.

Furthermore, the outcomes from the XGBoost (XGB) model were presented, offering appropriate variable rankings to elucidate the nexus between discernible features and ECG-Hb. Multivariate Cox proportional hazard models were used to investigate the association between ECG-Hb levels and outcomes. Comparative metrics include hazard ratios, each accompanied by a 95% confidence interval.

## Results

Table 1 shows the distribution of Hb levels, fundamental demographics, and historical disease data across the development, tuning, internal validation, and external validation sets. The average ages for these sets are 58.4 ± 18.5, 64.0 ± 17.7, 52.6 ± 17.8, and 57.6 ± 20.6 years, respectively. Among these cohorts, 53.4%, 50.7%, 53.4%, and 50.3% were male. The proportion of individuals with severe anemia (Hb ≤ 8) in these sets is 12.4%, 17.0%, 5.4%, and 7.2%, respectively. Meanwhile, the prevalence of moderate anemia (8 < Hb ≤ 10) in each set is 17.8%, 22.1%, 12.6%, and 15.4%, respectively.

**Table 1:** Baseline characteristics

	Development set	Tuning set	Internal validation set	External validation set
Hemoglobin profile				
Hb (g/dL)	12.9 ± 2.3	12.5 ± 2.4	13.6 ± 2.0	13.3 ± 2.1
Hb ≤ 8	39,568(12.4%)	11,879(17.0%)	1303(5.4%)	2120(7.2%)
8 < Hb ≤ 10	56,488(17.8%)	15,477(22.1%)	3066(12.6%)	4498(15.4%)
10 < Hb	222,026(69.8%)	42,698(61.0%)	19,910(82.0%)	22,629(77.4%)
Demography				
Sex (male)	169,991(53.4%)	35,506(50.7%)	12,974(53.4%)	14,702(50.3%)
Age (years)	58.4 ± 18.5	64.0 ± 17.7	52.6 ± 17.8	57.6 ± 20.6
BMI (kg/m^2^)	24.3 ± 4.2	24.2 ± 4.3	24.3 ± 4.1	24.1 ± 4.2
SBP (mmHg)	133.7 ± 26.9	138.6 ± 28.4	133.9 ± 26.5	137.8 ± 27.5
DBP (mmHg)	78.7 ± 16.8	78.0 ± 17.7	79.1 ± 16.3	77.5 ± 17.1
Disease history				
DM	57,918(18.2%)	21,563(30.8%)	3313(13.6%)	5726(19.6%)
HTN	88,605(27.9%)	34,048(48.6%)	6122(25.2%)	10,191(34.8%)
HLP	75,594(23.8%)	27,822(39.7%)	5015(20.7%)	8376(28.6%)
CKD	59,654(18.8%)	25,383(36.2%)	2014(8.3%)	3587(12.3%)
AMI	9825(3.1%)	4235(6.0%)	258(1.1%)	289(1.0%)
STK	33,534(10.5%)	13,244(18.9%)	1733(7.1%)	3209(11.0%)
CAD	53,596(16.8%)	22,089(31.5%)	3154(13.0%)	4855(16.6%)
HF	22,158(7.0%)	10,475(15.0%)	1038(4.3%)	1661(5.7%)
AF	12,175(3.8%)	6424(9.2%)	521(2.1%)	821(2.8%)
COPD	33,223(10.4%)	13,764(19.6%)	2239(9.2%)	4362(14.9%)

Hb, hemoglobin; BMI, body mass index; SBP, systolic blood pressure; DBP, diastolic blood pressure; DM, diabetes mellitus; HTN, hypertension; HLP, hyperlipidemia; CKD, chronic kidney disease; AMI, acute myocardial infarction; STK, stroke; CAD, coronary artery disease; HF, heart failure; AF, atrial fibrillation; COPD, chronic obstructive pulmonary disease

**Fig. 2:**
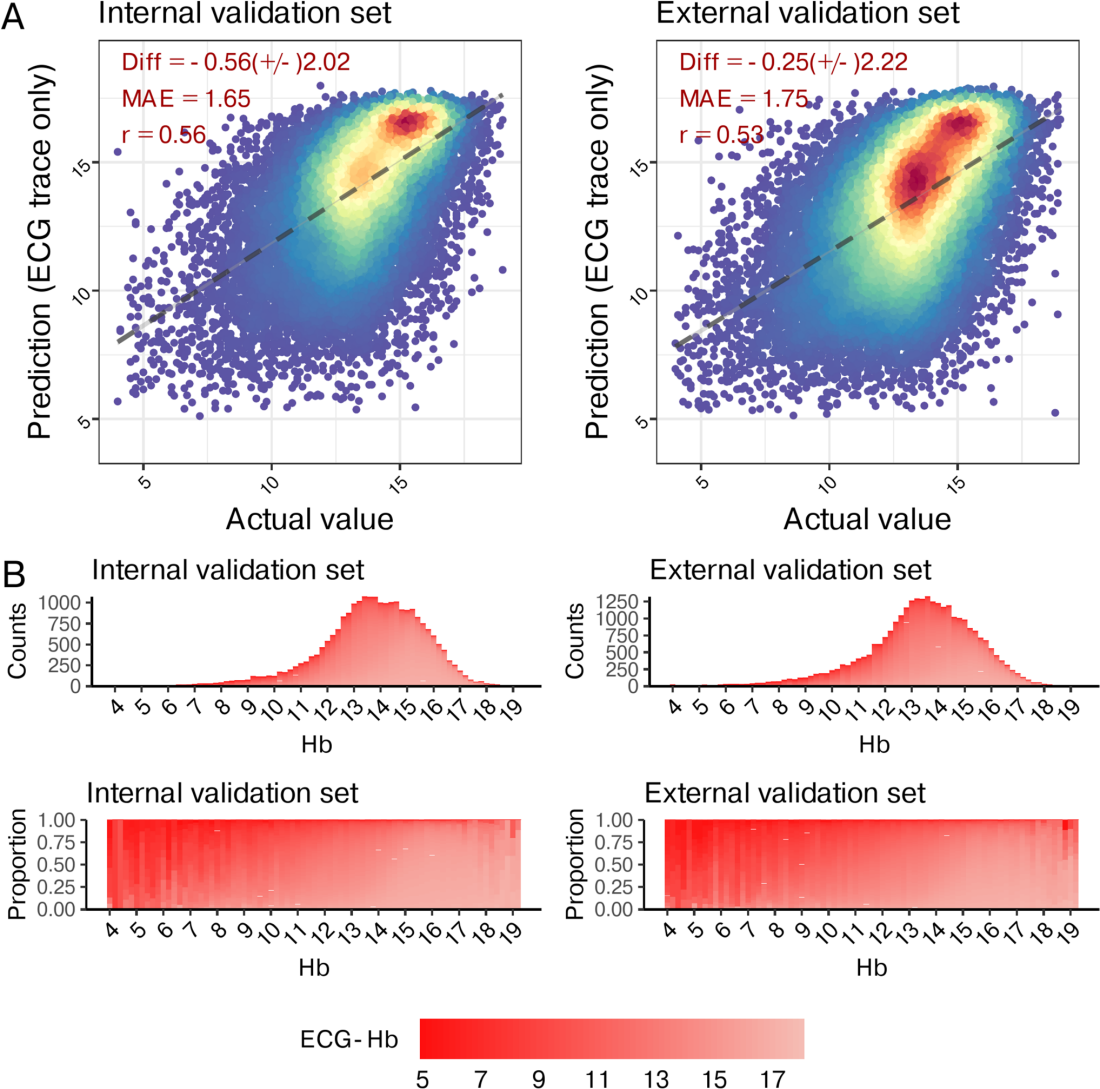
Predicted hemoglobin (ECG-Hb) and actual Hb. A) Scatter plots of ECG-Hb via ECG only compared to the actual Hb. The x-axis indicates the actual Hb, and the y-axis presents the ECG-Hb. Red points represent the highest density, followed by yellow, green, light blue, and dark blue. We presented the mean difference (Diff), Pearson correlation coefficients (r), and mean absolute errors (MAE) to demonstrate the accuracy of DLM. The black lines with 95% conference intervals are fitted via simple linear regression. B) The distributions of Hb in the internal and external validation sets. The color gradient from white to red demonstrated the ECG-Hb levels from normal to low. The panel above shows the original distribution of each dataset, and the panel below shows the distribution of ECG-Hb in each actual Hb value

Figure 2a shows a scatter plot comparing laboratory hemoglobin (Lab-Hb) to ECG-Hb. In the internal validation set, the MAE between Lab-Hb and ECG-Hb was 1.65, exhibiting a correlation coefficient of 0.56 and a Diff of -0.56 ± 2.02. Notably, the accuracy marginally diminished in the external validation set, with an MAE of 1.75, a correlation coefficient of 0.53, and a Diff of -0.25 ± 2.22. The upper segment of Fig. 2b shows the actual Lab-Hb distribution in both internal and external sets. Most patients in the internal validation set had a lab-Hb range of 12–16 g/dL, and a similar distribution was observed in the external validation set. While there appears to be no overt distinction in the Lab-Hb distribution between the internal and external validation sets, it is imperative to note that the external validation set (22.6%) exhibits a higher proportion of patients with Lab-Hb of ≤ 10 compared to the internal validation set (18.0%). For a balanced representation, the distribution of ECG-Hb for each lab-Hb value was visualized, revealing comparable proportions in both validation sets, as shown in the lower segment of Fig. 2b. In conclusion, a discernible trend was observed: As lab-Hb decreased, a higher proportion exhibited a correspondingly lower ECG-Hb.

**Fig. 3:**
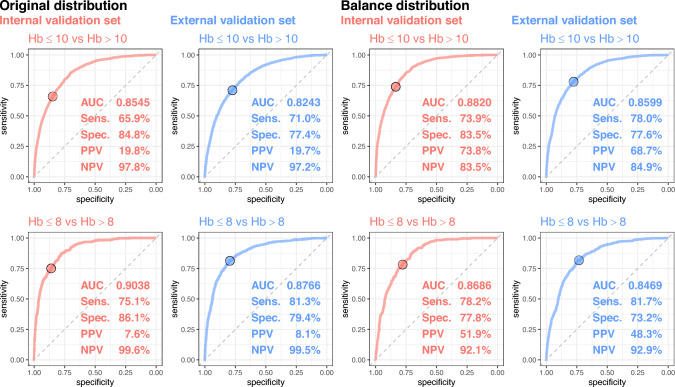
The ROC curve of DLM predictions based on ECG to detect moderate to severe anemia. Moderate and severe anemia were defined as actual hemoglobin (Hb) of ≤ 10 and ≤ 8, respectively. The operating point was selected based on the maximum of Youden’s index in the tuning set and presented using a circle mark, and the area under ROC curve (AUC), sensitivity (Sens.), specificity (Spec.), positive predictive value (PPV), and negative predictive value (NPV) were calculated based on it (left panel). Due to the different distributions of Hb in the internal and external validation sets, we generated the balance dataset for each set to ensure the same number of cases in different values of Hb (right panel)

Figure 3 (left panel) shows the proficiency of the DLM in identifying moderate and severe anemia. For the detection of moderate-to-severe anemia, the AUC was 0.8545, accompanied by a Sens of 65.9%, Spec of 84.8%, PPV of 19.8%, and NPV of 97.8%. Notably, the AUC was augmented to detect severe cases, registering 0.9038, with a Sens of 75.1% and a Spec of 86.1%. The raw accuracies in the external validation set declined slightly compared to those in the internal validation set. Specifically, for moderate anemia, the metrics were AUC = 0.8243, Sens = 71.0%, Spec = 77.4%, PPV = 19.7%, and NPV = 97.2%. The AUC for detecting severe cases (0.8766) was marginally reduced in the external set compared to that of the internal set. Considering the higher prevalence of moderate cases in the external validation set (22.6%) than in the internal validation set (18.0%), it may be more challenging for the DLM to differentiate severe cases from moderate ones compared to normal cases. Therefore, a balanced analysis was performed for each set, considering the respective distributions depicted in Fig. 3 (right panel). The AUCs for the external validation set (0.8599 for moderate anemia and 0.8469 for severe anemia) remained slightly lower than those of the internal validation set (0.8820 for moderate anemia and 0.8686 for severe anemia). This discrepancy might stem from variations in patient characteristics between the external and internal validation sets; it was not caused by the different distributions of Lab-Hb.

**Fig. 4:**
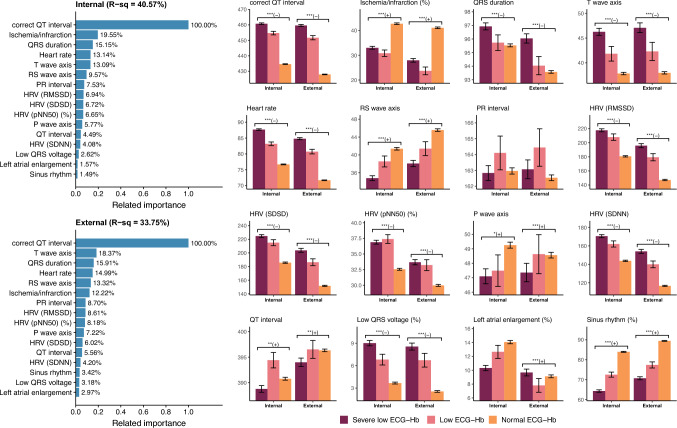
Relationship between selected ECG features and predicted hemoglobin (ECG-Hb). The related importance is based on the information gained by the XGB model, and the R-square (R-sq) is the coefficient of determination used in selected ECG features for predicting ECG-Hb. AI-ECG prediction was classified as normal ECG-Hb, low ECG-Hb, and severe low ECG-Hb based on the operating points, the same as the previous ROC curve analysis. The analyses were conducted both in the internal and external validation sets. (*: p for trend < 0.05; **: p for trend < 0.01; ***: p for trend < 0.001)

Extended Fig. 1 delineates the significance of the relationship between all ECG features and ECG-Hb as determined by the information gained from the XGB model. The coefficients of determination (R-square) values based on all ECG features were 40.90% and 34.33% for the internal and external validation sets, respectively. Figure 4 shows the 12 most noticeable ECG features pertinent to ECG-Hb. The R-square values derived from these features were congruent with those obtained using all the ECG features, registering 40.57% for the internal validation set and 33.75% for the external validation set. Within the internal validation set, the ECG features of paramount importance to ECG-Hb included corrected QT interval, ischemia/infarction markers, QRS duration, T-wave axis, heart rate, and RS-wave axis in descending order. Notably, ECG measurements and HRV played key roles in establishing ECG-Hb levels, which had more significance than ECG morphology. The top six features in the external validation set mirrored those of the internal set, albeit with a slight reordering. The corrected QT interval may be the most important ECG feature for establishing ECG-Hb. Specifically, the corrected QT interval was the longest in the severely low group, followed by the low group and the normal group.

**Fig. 5:**
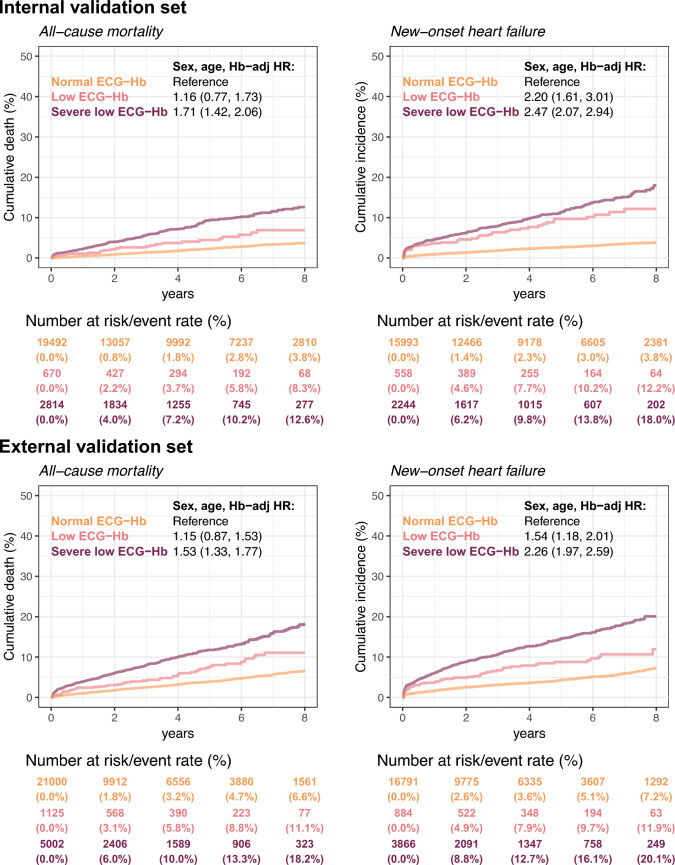
Long-term incidence of developing new-onset cardiovascular events in patients with an initially normal hemoglobin (Hb) of > 10 mg/dL stratified by AI-ECG prediction. Based on the operating points, AI-ECG prediction was classified as normal ECG-Hb, low ECG-Hb, and severe low ECG-Hb, the same as with the previous ROC curve analysis. The analyses were conducted both in internal and external validation sets. The table shows the at-risk population and cumulative risk for the given time intervals in each risk stratification

Figure 5 shows the disparity in new-onset cardiovascular events between false-positive and true-negative cases, underscoring the prognostic value of ECG-Hb levels. Within the internal validation set, the incidence of all-cause mortality in the severely low ECG-Hb group was 4.0% at 2 years and increased to 12.6% at 8 years, markedly elevated compared to the normal ECG-Hb group, which registered at 0.8% and 3.8%, respectively, yielding an adjusted HR of 1.71 (95% CI: 1.42–2.06). A conspicuous dose–response relationship was evident, transitioning from the HR of the low ECG-Hb group (1.16, 95% CI: 0.77–1.73) to that of the severe low ECG-Hb group. This correlation was corroborated using an external validation set. Further analysis on the onset of heart failure revealed HRs of 2.20 (95% CI: 1.61–3.01) and 2.47 (95% CI: 2.07–2.94) for the low ECG-Hb group and severe low ECG-Hb group, respectively, within the internal validation set. Notably, a pronounced dose–response relationship persisted. Parallel analyses conducted on the external validation set yielded outcomes consistent with those of the internal set, thereby emphasizing the ability of ECG-Hb to identify cardiovascular precursors.

## Discussion

This study presents and evaluates ECG-Hb as a novel approach for detecting anemia. The model demonstrated promising diagnostic accuracy with robust correlations between ECG-Hb and laboratory-based Hb levels, particularly in distinguishing between moderate and severe anemia. In addition, this study highlights the significance of specific ECG features in predicting Hb levels and offers insights into possible mechanistic pathways. Notably, ECG-Hb exhibits prognostic significance as it is associated with all-cause mortality and the incidence of heart failure.

Compared to a previous study with an AUC exceeding 0.85 [29], our approach achieved a comparable performance in anemia detection, with AUCs surpassing 0.82. This discrepancy stems from the distribution of Hb levels. A previous study was conducted in a population with an average Hb of 13.5 g/dL and an anemia prevalence of merely 4–5% [29], contrasting sharply with our study's 18–22% prevalence. It is more challenging to differentiate between Hb levels of 9 g/dL and 11 g/dL than between those of 9 and 15 g/dL. Thus, our accuracy is similar to that of previous reports. Notably, our accuracy is not inferior to that of widely implemented screening tests, such as breast cancer screening (AUC = 0.78)[38] and fecal occult blood tests (AUC = 0.71) [39]. Given the cost-effectiveness, ubiquity, and frequent use of ECG, AI-ECG can potentially detect asymptomatic anemia with satisfactory precision. This AI model is particularly suited for anemia screening in resource-limited regions that may lack standardized testing procedures and equipment. Reports indicate anemia prevalence rates as high as 30% in women from these areas [1], making our study potentially more representative of real-world applications.

Our study determined that AI-ECG primarily relies on ECG and HRV parameters when estimating ECG-Hb levels. Anemia appears to prolong the QT interval, potentially due to subendocardial ischemia resulting from a demand/supply mismatch in the coronary physiology of anemic individuals [40]. Moreover, past studies have found that barring high-frequency power, each 1 g/dL decrease in hemoglobin correlated with increased odds of low HRV [41]. Additionally, anemia elevates heart rate, attributed to hypoxia-stimulated chemoreceptors and heightened sympathetic activity [42]. These evidence and our findings suggest a potential correlation between ECG and anemia, although physicians may struggle to discern anemia from these subtle signs. With the advancement of deep learning, its capability to uncover unknown correlations has been validated [43]. Our study confirms the relevance of deep learning in predicting Hb from ECG, suggesting potential for future exploration of ECG's relationship with other diseases using this technology.

The primary advancement of this study over prior research lies in introducing a continuous ECG-Hb measure to assess the severity of anemia rather than relying on probability outputs from traditional machine learning models. This continuous measure provides improved interpretability of disease severity. However, note that ECG-Hb is not a direct substitute for Lab-Hb. Despite their moderate to high correlation (r = 0.53–0.56), understanding discrepancies, especially false positives, is vital. Our analysis revealed that while the NPV of our AI model consistently exceeded 97%, the PPV was considerably lower (19.7–19.8%). We identified a significant association between false-positive results and anemia-related complications. This aligns with previous findings, suggesting that AI-ECG is capable of pinpointing cardiovascular previvors [44]. Furthermore, our analysis indicated that positive AI-ECG predictions stemmed from a series of ECG changes. Patients with abnormal ECGs are at an elevated risk of cardiovascular disease. Beyond the heart changes indicated by abnormal ECGs, certain rhythms may require intervention. For instance, atrial fibrillation is linked to an increased stroke risk and requires treatment [45]. Given the potential benefits of early detection and management, physicians should prioritize positive AI-ECG predictions, extending beyond just anemia.

Globally, areas with the most significant prevalence of anemia are mostly those with substantial resource limitations [1]. ECGs are considerably more affordable and user-friendly than blood analysis machines, making them ideal for anemia screening in these areas. With the aid of AI, ECGs can simultaneously detect multiple diseases [44], offering cost-effective large-scale screening in resource-limited regions. Despite the ubiquity of blood tests in developed countries, over three million ECGs are conducted daily worldwide [46]. Consequently, some patients with anemia may only undergo ECGs without blood test verification. Opportunistic screening, an emerging concept originating from the incidental discovery of "incidentalomas" in radiology [47], has gained traction [48]. Radiologists have embraced AI models that re-analyze radiological images to identify potential diseases. Likewise, ECGs offer a similar potential [49]. A randomized controlled trial has shown that opportunistic AI-ECG analysis can identify an additional 32% of patients with left ventricular dysfunction [50]. Future research should explore AI-ECG's potential to detect more asymptomatic anemic patients, facilitating early diagnosis and intervention to improve patient outcomes.

Although this study presents promising findings regarding the development and evaluation of ECG-Hb for the detection of anemia, it is essential to acknowledge certain limitations. First, we used retrospective data from two specific hospitals, which may limit the generalizability of the model to broader patient populations and healthcare settings. The differences in inclusion periods and baseline characteristics among the cohorts used for model training, tuning, and validation could have influenced the observed prevalence of HRV measures and ECG patterns. Additionally, this study primarily focused on the relationship between ECG-Hb and anemia, emphasizing diagnostic accuracy and prognostic value. Potential confounding variables and underlying mechanisms, such as systemic conditions affecting autonomic nervous system, QT interval or HRV, that may contribute to the observed associations were not explored. Furthermore, while ECG-Hb demonstrates promising results in anemia detection and its implications for cardiovascular outcomes, the practical implementation of this model in real-world clinical practice and resource-constrained regions requires further validation. Future studies should assess its cost-effectiveness, scalability, and sustainability, as the financial and technical resources required to establish and maintain such an AI system must be carefully evaluated. Moreover, the current study does not allow a direct comparison between the AI-ECG model and conventional clinical evaluation, including history taking, physical examination, or longitudinal ECG assessment. Integrating serial ECG changes or combining AI-ECG analysis with clinical evaluation and historical data could further enhance diagnostic performance and prognostic capabilities. Finally, this study relied on historical data. The rapidly evolving field of deep learning and big data healthcare may necessitate ongoing validation and adaptation of the ECG-Hb model to ensure its relevance and efficacy in the ever-changing healthcare landscape.

## Conclusion

ECG-Hb is useful for detecting anemia and cardiovascular risk factors. The model showed the efficacy of ECG-based biomarkers in anemia identification, underlined by a robust correlation with lab-measured Hb levels. Its diagnostic precision, especially in discerning moderate-to-severe anemia, and its prognostic relevance linked to mortality and heart failure onset highlight the transformative potential of AI in disease screening, especially in resource-limited settings. Future studies should explore its practical deployment and physiological underpinnings to maximize its clinical applications.

## Supplementary Information

Below is the link to the electronic supplementary material.

Supplementary file 1 (PDF 309 KB)
