# Supplementary material for: Non-invasive hemoglobin estimation with outcome prediction via deep learning analysis of ECG-derived cardiac micro-dynamics
Source: Intern Emerg Med. 2025 Dec 2;21(2):525–36. doi: 10.1007/s11739-025-04209-6 (PMC13061774; doi:10.1007/s11739-025-04209-6)
Supplement: Supplementary file 1 — (PDF 309 KB) [file 11739_2025_4209_MOESM1_ESM.pdf]

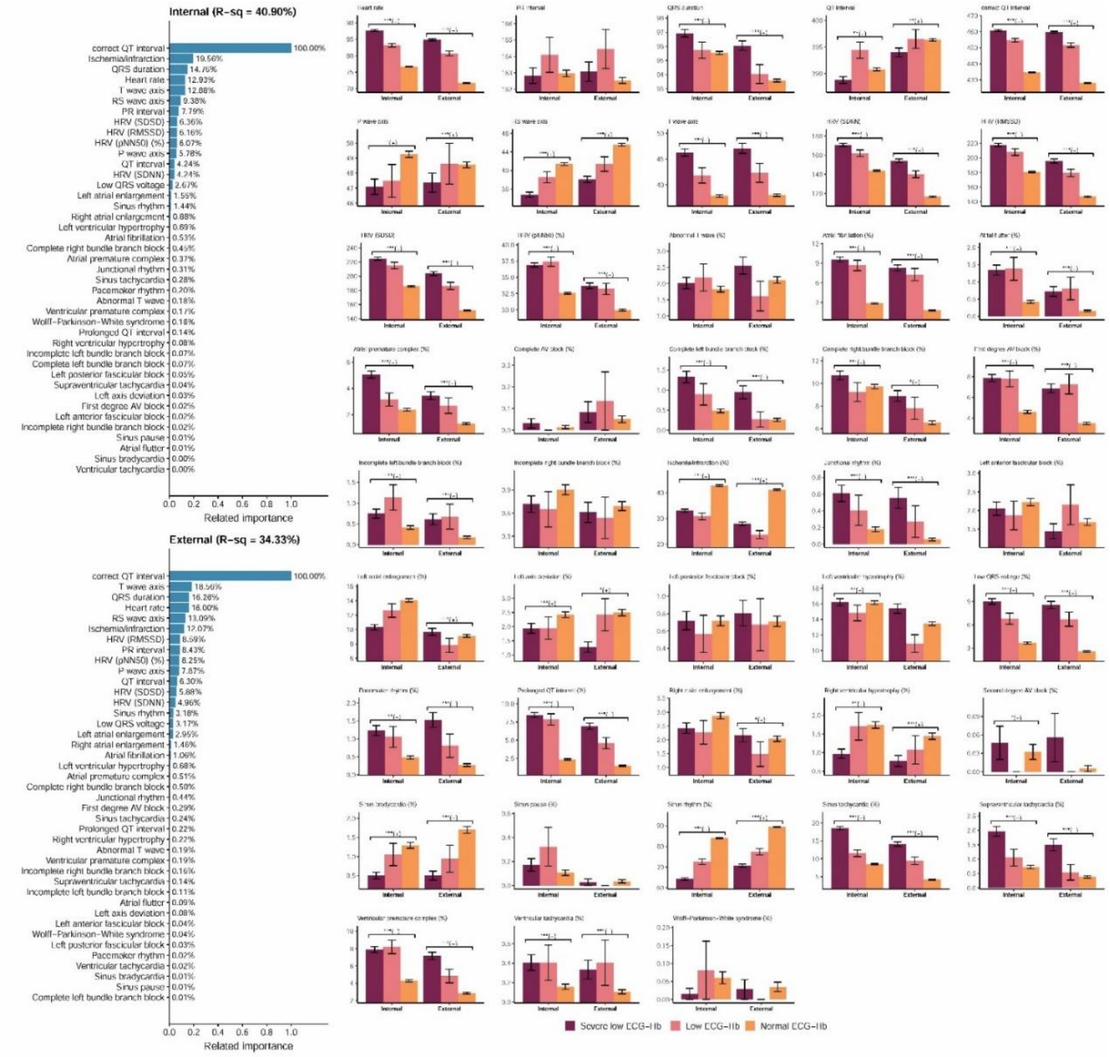

Extend Fig. 1 Relationship between all ECG features and predicted hemoglobin (ECG-Hb). The related importance is based on the information gained from the XGB model and the R-square (R-sq), which is the coefficient of determination for using all ECG features to predict ECG-Hb. AI-ECG prediction was classified as normal ECG-Hb, low ECG-Hb, or severely low ECG-Hb, based on the operating points, similar to the previous ROC curve analysis. Analyses were conducted using both internal and external validation sets. (*: p for trend < 0.05; **: p for trend < 0.01; ***: p for trend < 0.001).
